# Validation of fluence‐based 3D IMRT dose reconstruction on a heterogeneous anthropomorphic phantom using Monte Carlo simulation

**DOI:** 10.1120/jacmp.v16i1.5199

**Published:** 2015-01-08

**Authors:** Yuji Nakaguchi, Takeshi Ono, Masato Maruyama, Nozomu Nagasue, Yoshinobu Shimohigashi, Yudai Kai

**Affiliations:** ^1^ Department of Radiological Technology Kumamoto University Hospital Kumamoto Japan; ^2^ Faculty of Life Sciences Kumamoto University Kumamoto Japan

**Keywords:** quality assurance, verification, three‐dimensional measurement, IMRT, three‐dimensional reconstruction

## Abstract

In this study, we evaluated the performance of a three‐dimensional (3D) dose verification system, COMPASS version 3, which has a dedicated beam models and dose calculation engine. It was possible to reconstruct the 3D dose distributions in patient anatomy based on the measured fluence using the MatriXX 2D array. The COMPASS system was compared with Monte Carlo simulation (MC), glass rod dosimeter (GRD), and 3DVH, using an anthropomorphic phantom for intensity‐modulated radiation therapy (IMRT) dose verification in clinical neck cases. The GRD measurements agreed with the MC within 5% at most measurement points. In addition, most points for COMPASS and 3DVH also agreed with the MC within 5%. The COMPASS system showed better results than 3DVH for dose profiles due to individual adjustments, such as beam modeling for each linac. Regarding the dose‐volume histograms, there were no large differences between MC, analytical anisotropic algorithm (AAA) in Eclipse treatment planning system (TPS), 3DVH, and the COMPASS system. However, AAA underestimated the dose to the clinical target volume and Rt‐Parotid slightly. This is because AAA has some problems with dose calculation accuracy. Our results indicated that the COMPASS system offers highly accurate 3D dose calculation for clinical IMRT quality assurance. Also, the COMPASS system will be useful as a commissioning tool in routine clinical practice for TPS.

PACS number: 87.55.Qr, 87.56.Fc, 87.61.Bj

## I. INTRODUCTION

In patient‐specific intensity‐modulated radiation therapy (IMRT) quality assurance (QA), ion chamber measurement and film measurement have traditionally been performed in homogeneous phantoms. This method provides useful empirical evidence,^(1)^ but suffers from the potential failure to record the dose from parts of each beam in that do not intersect the film plane. Another common method is two‐dimensional (2D) arrays which measure by each beam in a homogeneous phantom. Recent publications^2^, ^3^ have argued that beam‐by‐beam and homogeneous phantom measurements for IMRT QA can be misleading and insensitive to dosimetric errors. A three‐dimensional (3D) measurement system in the patient is needed for accurate dose verification.

Recently, IBA Dosimetry (IBA Dosimetry, GmbH, Germany) released the 3D measurement system COMPASS version 3. The COMPASS system consists of the MatriXX 2D array^(4)^ based on pixel ionization chamber mounted on a linear accelerator head and an integrated software solution comprising an algorithm that models the linear accelerator head and detector. It is possible to reconstruct the 3D dose distribution in the patient based on fluence measured with the MatriXX.

Boggula et al.^5^, ^6^ applied the dose verification with the COMPASS system for IMRT and VMAT plans. The reconstructed 3D dose distributions from the COMPASS system were compared with that from a treatment planning system (TPS) and the measured dose distributions using the MatriXX detector and EDR2 films (Eastman Kodak, Rochester, NY) in homogeneous phantoms. Korevaar et al.^(7)^ also demonstrated the performance of the COMPASS system using a multileaf collimator (MLC) test pattern and a clinical case on a homogeneous phantom. They concluded that the agreement between the measurements and the dose reconstruction from the COMPASS depends on the treatment modality, as well as on the target shape.

However, these previous studies did not demonstrate validation using an anthropomorphic phantom. Furthermore, we need a simulation such as Monte Carlo simulation (MC) for reliable evaluation.

The purpose of the present study was to investigate the accuracy of reconstructed 3D dose distributions from the new version of the COMPASS system using an anthropomorphic phantom for clinical IMRT planning. The dose distributions were compared with those of another 3D verification system, glass rod dosimeter (GRD) (Asahi Techno Glass Corporation, Shizuoka, Japan), and MC.

## II. MATERIALS AND METHODS

### A. The COMPASS system

**Figure 1:**
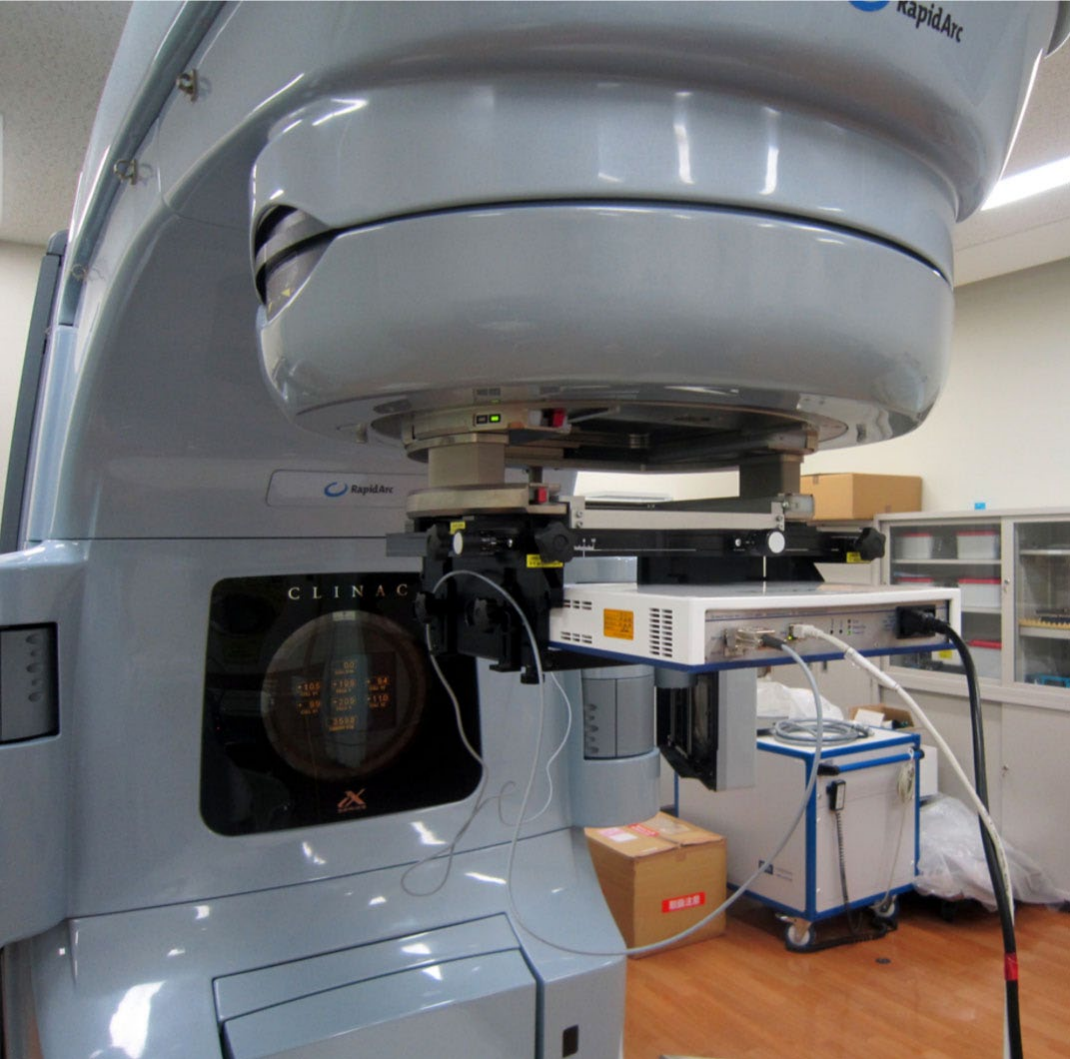
The MatriXX detector mounted on the gantry of a linear accelerator.

The COMPASS system consists of the MatriXX 2D array based on a pixel ionization chamber, an integrated software solution comprising an algorithm which models the linear accelerator head and detector, and an angle sensor. The detector assembly was mounted in a holder attached to the treatment head of a Varian Clinac iX (Varian Medical Systems, Palo Alto, CA) linear accelerator with a source‐to‐detector distance of 76.2 cm (see Fig. 1). The MatriXX has 1020 ion chambers at intervals of 7.6 mm^4^. On top of the detector, the solid water slabs (RMI‐457, GAMMEX, GmbH, Germany) of 2 cm thickness were used for extra buildup and removal of electron contamination.

The measurement for the COMPASS system is fluence measurement. We measure fluence from the linear accelerator head using the Matrixx detector. The COMPASS can then calculate the 3D dose distribution from measured fluence, TPS data (images, structures, plan, dose), and beam modeling data. The measured fluence is applied to a superposition formula.^(8)^ The superposition algorithm can generate the 3D dose distribution from 2D fluence data. We checked the beam modeling for the COMPASS system using open simple fields.

### B. Phantom

**Figure 2:**
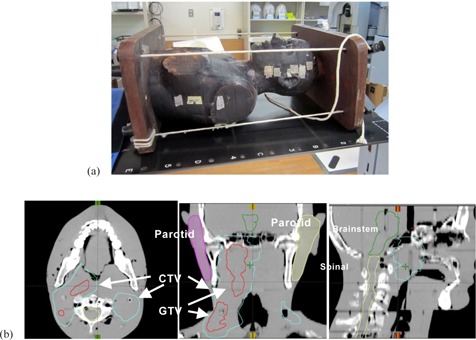
RANDO Alderson phantom (a) and contouring of target and OAR on TPS (b).

The anthropomorphic phantom used in this work was a RANDO Alderson (Radiology Support Devices Inc., Long Beach, CA), as shown in Fig. 2(a). To prepare the treatment plan, the head and neck area was scanned using a CT scanner (LightSpeed RT, GE Healthcare, Little Chalfont, UK). The slice thickness was 2.5 mm. Next, we made contours of the critical organs and the target assuming a nasopharyngeal carcinoma. The extracted organs were the brain, brainstem, spinal cord, eyes, lens, optic nerves, chiasm, parotid, mandible, and larynx. Figure 2(b) shows some extracted structures.

### C. Treatment planning

We performed head and neck (H&N) IMRT treatment planning, consisting of 7 static beams using Eclipse TPS (Varian Medical Systems). The X‐ray energy and prescribed dose were 6 MV and 34 Gy (200 cGy/17 fraction) for clinical target volume (CTV). The calculation grid size was $2.5\times 2.5\times 2.5\;{\text{mm}}^{3}$. Table 1 shows our summary of target coverage and organ‐at‐risk (OAR) constraints.

**Table 1:** Summary of target coverage and OAR constraints required for the anthropomorphic RANDO Alderson phantom with the planning/delivery technique

Planning Delivery Technique	6 MV, 7 beams static IMRT	
Prescription/Target Coverage	34 Gy / 17 fractions for CTV	
	Maximum dose for CTV	<110%dose(Gy)
	95% volume for CTV	>95%dose(Gy)
	Volume of CTV receives 93% dose	>99%
OAR Constraints	Maximum Spinal Cord	<45Gy
	Maximum Brain Stem	<54Gy
	Maximum Mandible	<65Gy
	Maximum Chiasm	<54Gy
	Maximum Larynx	<40Gy
	Maximum Optic Nerve (Rt, Lt)	<54Gy
	Maximum Eye (Rt, Lt)	<40Gy
	Maximum Lens (Rt, Lt)	<10Gy
	Parotid (Rt, Lt)	<25Gy of 50% vol.

### D. Point‐doses measurement

**Figure 3:**
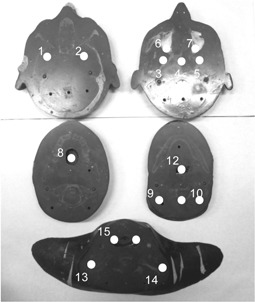
Measurement points for GRD in RANDO Alderson phantom. We inserted GRD in the cavities of the RANDO Alderson phantom and measured them at 1–16 measurement points.

The dose measurements in the anthropomorphic RANDO Alderson phantom were performed using the GRD detectors at 16 selected positions in the H&N section of this phantom, as shown in Fig. 3. The diameter and length of the GRD detector were 1.5 mm and 8.5 mm. The GRD detectors were calibrated using a 6 MV photon beam in a solid water phantom at a depth of 10 cm. The detailed procedure of GRD measurements is described elsewhere.^(9)^

### E. MC simulation

**Figure 4:**
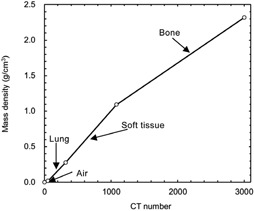
Conversion curve of CT number to materials and mass density.

To verify the accuracy of the COMPASS system, the dose profiles and the dose distribution for head and neck (H&N) planning were also calculated by the EGSnrc /BEAMnrc^10^, ^11^ and DOSXYZnrc^(12)^ user codes. Incident photon particles were derived from the treatment‐head simulations with a 6 MV photon beam for a Varian Clinac iX. In MC calculations for the H&N plan, a voxel‐based phantom was used. The voxel‐based phantom was created by conversion of CT images into materials (air, lung, soft tissue, and bone) and mass densities. The conversion curve of the CT number to materials and mass density is shown in Fig. 4. In MC dose calibration, we calculated the absorbed dose to water, $D_{\text{MC}}\;[\text{Gy}/\text{particle}]$ at 10 cm depth in 30×30×30cm water phantom using MC simulation at the beginning. Next, we measured the absorbed dose to water per MU, $D_{\text{meas}}\;[\text{Gy}/\text{MU}]$ at the same point. Finally, we obtained the calibration factor from the following equation:

(1) $$\text{CF}=D_{\text{meas}}/D_{\text{MC}}[\text{MU}/\text{particle}]$$

The total number of histories is $1.0\times 10^{4}$ in MC dose to water calculation and multiplied by the same monitor units on the TPS for each treatment field. The calculation grid size was $3.0\times 3.0\times 2.5\;{\text{mm}}^{3}$. The energy threshold and cutoff were AE=ECUT=0.7MeV and AP=PCUT=0.01MeV.

### F. Comparison to other 3D measurement systems

We compared the COMPASS system with other 3D measurement using 3DVH^2^, ^3^ (Sun Nuclear Corp., Melbourne, FL). First, we irradiated the ArcCHECK phantom to acquire the measurement data. Next, we exported the Digital Imaging and Communication in Medicine (DICOM) files (RT plan, RT dose, RT structures) and CT images from the TPS to 3DVH. The DICOM files and the measurement data from the ArcCHECK phantom were used to generate dose‐volume histograms (DVHs) and profiles for an anthropomorphic RANDO Alderson phantom.

## III. RESULTS & DISCUSSION

### A. Point‐dose measurement

Table 2 shows the results of point‐dose measurements on the anthropomorphic RANDO Alderson phantom. The maximum difference between MC and GRD was 11% at measurement point 8. However, most of the GRD measurement points agreed with the MC within 5%. The accuracy of GRD measurement was almost 5% for the phantom measurement and was consistent with the previous study.^(13)^ Furthermore, we have to consider the angular dependence and geometric uncertainties for GRD measurement in the anthropomorphic phantom because the cavities for measurement in the phantom are larger than the size of GRD. Therefore, the difference between MC and GRD measurement is within the measurement uncertainty for GRD measurement. This result shows that it is acceptable to use MC as a reference. Meanwhile, the results of TPS show good agreement with MC and confirm that this IMRT plan does not have any problems in terms of accuracy at the measurement points. In addition, the 3DVH and COMPASS reconstructed patient dose is generally close to MC. The maximum difference between MC and COMPASS was 9%. Most points were agreement with the MC within 5%. As for point doses, there were no clear differences between MC, 3DVH, and COMPASS.

**Table 2:** Comparison of point doses in the anthropomorphic RANDO Alderson phantom between MC, GRD, TPS, 3DVH, and COMPASS at measurement points 1–16

*Measurement Points*	*MC cGy*	*GRD*		*TPS*		*3DVH*		*COMPASS*		*SD cGy*
		*cGy*	*RGD/MC*	*cGy*	*TPS/MC*	*cGy*	*3DVH/MC*	*cGy*	*COMPASS/MC*	
1	114.3	116.9	1.02	113.3	0.99	113.5	0.99	111.2	0.97	2.06
2	205.0	214.1	1.04	205.1	1.00	202.6	0.99	205.3	1.00	4.43
3	140.3	151.2	1.08	144.0	1.03	139.6	0.99	141.0	1.00	4.77
4	201.9	196.3	0.97	202.9	1.00	198.5	0.98	205.7	1.02	3.70
5	205.2	204.7	1.00	202.1	0.98	205.0	1.00	204.8	1.00	1.28
6	114.3	111.1	0.97	112.3	0.98	112.7	0.99	114.8	1.00	1.52
7	194.5	197.8	1.02	198.0	1.02	196.5	1.01	197.5	1.02	1.43
8	117.4	130.2	1.11	131.1	1.12	125.9	1.07	127.8	1.09	5.48
9	165.8	170.5	1.03	173.5	1.05	168.5	1.02	170.5	1.03	2.86
10	142.6	151.5	1.06	145.1	1.02	150.0	1.05	148.7	1.04	3.66
11	189.4	187.0	0.99	190.7	1.01	189.4	1.00	191.0	1.01	1.58
12	195.0	189.5	0.97	200.2	1.03	196.5	1.01	199.6	1.02	4.30
13	132.4	138.5	1.05	139.2	1.05	137.5	1.04	137.9	1.04	2.70
14	150.2	152.1	1.01	155.8	1.04	152.5	1.01	152.0	1.01	2.03
15	163.0	157.4	0.97	154.6	0.95	156.7	0.96	156.4	0.96	3.16
16	176.7	178.6	1.01	174.5	0.99	178.0	1.01	176.2	1.00	1.60

SD=standard deviation.

### B. Comparison of dose profiles

**Figure 5:**
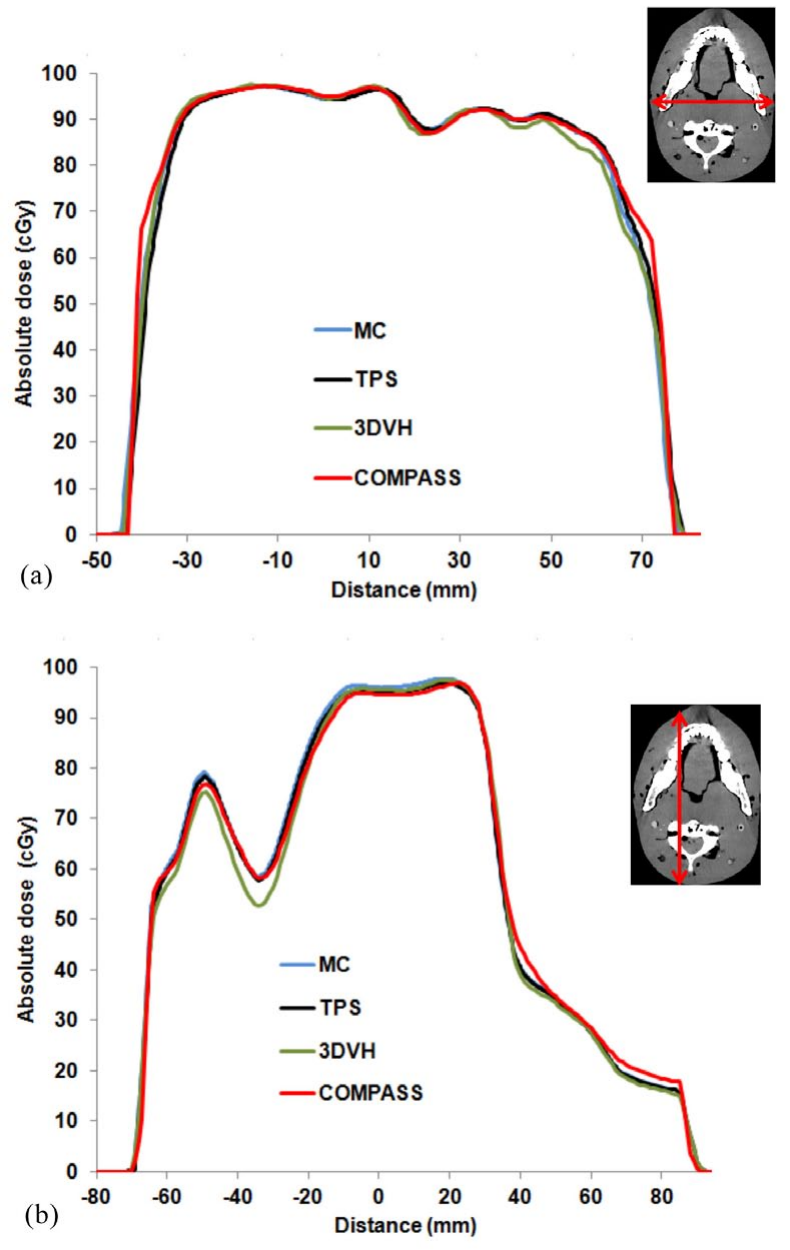
Comparison of IMRT dose profiles between TPS, 3DVH, COMPASS, and MC calculations at an isocenter: (a) lateral direction on the axial image at the isocenter; (b) vertical direction on the axial image at the isocenter.

Figure 5 shows dose profiles at an isocenter for the RANDO Alderson phantom. The dose profiles reconstructed from COMPASS were almost in agreement with the MC‐calculated dose profiles. Again, there were no clear differences between MC, 3DVH, and COMPASS. However, 3DVH showed a little difference from MC and COMPASS in the high‐dose gradient area (see Fig. 5(b)). Watanabe and Nakaguchi^(14)^ reported some limitations of 3DVH. 3DVH has around 3% of measurement uncertainty, including systematic error. 3DVH does not request individual adjustments, such as beam modeling and customized CT‐relative electron density table, for each linac system. However, these simple measurement processes may create some uncertainties. As for resolution, COMPASS shows good spatial resolution in Fig. 5. Godart et al.^(8)^ also demonstrated the capability of the system to detect MLC leaf position errors using the COMPASS system. The detection of MLC errors requires a high‐resolution detector because most MLC errors are only a few millimeters. The COMPASS system has a resolution of a few millimeters, which is smaller than the size of the detector.

### C. The comparison of DVHs and gamma analysis

First, we compared between the COMPASS, 3DVH, and MC by 3D gamma analysis for a 3D evaluation. The gamma pass rate was performed with criteria of 2 mm and 3%, which are distance‐to‐agreement and percent dose agreement, respectively. The pass rate for body structure of the COMPASS, 3DVH, and MC were 96.2%, 95.1%, and 94.6%, respectively. There were no clear differences between the COMPASS, 3DVH, and MC.

**Figure 6:**
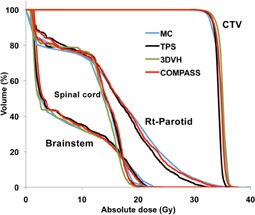
Comparison of DVHs in anthropomorphic RANDO Alderson phantom between COMPASS, 3DVH, TPS, and MC for neck plan.

Figure 6 and Table 3 show a comparison of DVHs in the anthropomorphic RANDO Alderson phantom between the COMPASS, 3DVH, and MC. The 3DVH showed the largest differences (7%, see Table 3) from MC. Those results are similar to the results of dose profiles, demonstrating the limitation of 3DVH whereby it does not need to be customized for each linac. TPS also underestimated the CTV and Rt‐Parotid slightly. This is because the AAA equipped with Eclipse TPS has some issues with dose calculation accuracy.^(15)^ In contrast, the COMPASS is equipped with superposition as a calculation engine and showed a calculation accuracy equal to that of MC in this study. Chow et al.^(16)^ also found that the accuracy of the superposition algorithm is the closest to MC, except in low density regions such as the lung. For the point measurement, TPS showed sufficient accuracy, but showed slight differences from other methods in DVHs. Carrasco et al.^(2)^ demonstrated the differences between 2D evaluation and 3D evaluation, and argued for the importance of 3D evaluation. This is because 2D evaluation is only a sampling technique for a treatment plan. The point‐dose evaluation is the same in 2D evaluation, and is only a sampling method for 3D dose distribution. We need a comparison using 3D evaluation for the accurate evaluation of treatment plans.

**Table 3:** Comparison of DVHs parameters in the anthropomorphic RANDO Alderson phantom between MC, TPS, 3DVH, and COMPASS

			*TPS*		*3DVH*		*COMPASS*	
*Structure*	*Constraints*	*MC*		*TPS/MC*		*3DVH/MC*		*COMPASS/MC*
CTV	Maximum dose (Gy)	38.0	36.4	0.96	37.5	0.99	37.3	0.98
	D95 (Gy)	33.4	33.1	0.99	33.6	1.01	33.2	0.99
Rt‐Parotid	D50 (Gy)	17.1	16.3	0.95	16.6	0.97	17.4	1.02
Spinal Cord	Maximum dose (Gy)	21.2	20.5	0.97	19.7	0.93	20.1	0.95
Brainstem	Maximum dose (Gy)	23.1	22.2	0.96	21.8	0.94	22.0	0.95

In this study, we can confirm the accuracy, including the resolution, of the COMPASS system for IMRT QA tool. In addition, this system provides rapid 3D dose verification because the COMPASS QA process does not require phantom plans, and therefore it simplifies the QA workflow. The COMPASS system contributes to fast and reliable 3D dose verification for clinical IMRT QA.

Meanwhile, commissioning for TPS requires verification using 3D measurement, including DVHs. COMPASS was originally a verification tool for IMRT, but the high accuracy of dose calculation for COMPASS makes it possible to use it for TPS commissioning. TPS commissioning is an important issue for us; we need a simple and reliable comprehensive commissioning method for TPS. COMPASS provides a new commissioning technique using fluence‐based 3D dose reconstruction. However, the verification for commissioning tool is insufficient for only this study. We need more verification using more treatment plans involving different sites (e.g., lung and pelvis).

## IV. CONCLUSIONS

In this study, we evaluated the accuracy of reconstructed dose distributions from the COMPASS system in an anthropomorphic phantom by using a complicated IMRT neck plan. The physical resolution of the COMPASS detector was lower than those of TPS and MC, but the dose resolution for dose profiles was comparable to TPS and MC by dose interpolation. The accuracy of reconstructed dose distributions from the COMPASS system was higher than 3DVH and the same as MC. Also, the COMPASS system will be a useful commissioning tool in routine clinical practice for TPS.

## ACKNOWLEDGMENTS

The authors wish to thank Mr. Lin Xu (IBA Dosimetry, Schwarzenbruck, Germany), Mr. Shunji Saiga (Toyo‐medic, Fukuoka, Japan), and Mr. Yasuo Takanashi (Toyo‐medic, Tokyo, Japan) for their help and discussion during this work.
